# Supplementary material for: Time-series transcriptome analysis identified differentially expressed genes in broiler chicken infected with mixed Eimeria species
Source: Front Genet. 2022 Aug 8;13:886781. doi: 10.3389/fgene.2022.886781 (PMC9393255; doi:10.3389/fgene.2022.886781)
Supplement: Supplementary file 2 [file DataSheet1.ZIP › 7dpi_GO.Gsea.1625106495923/GOBP_POSITIVE_REGULATION_OF_GLYCOLYTIC_PROCESS.html]

Details for gene set GOBP\_POSITIVE\_REGULATION\_OF\_GLYCOLYTIC\_PROCESS[GSEA]

|  || Dataset | TMM\_7dpi\_gct\_format\_7dpi\_gct\_format.Class\_4dpi.cls #PC\_versus\_NC.Class\_4dpi.cls #PC\_versus\_NC\_repos |
| Phenotype | Class\_4dpi.cls#PC\_versus\_NC\_repos |
| Upregulated in class | 0 |
| GeneSet | GOBP\_POSITIVE\_REGULATION\_OF\_GLYCOLYTIC\_PROCESS |
| Enrichment Score (ES) | -0.69640374 |
| Normalized Enrichment Score (NES) | -1.8575704 |
| Nominal p-value | 5.053057E-4 |
| FDR q-value | 0.09607787 |
| FWER p-Value | 0.703 |
Table: GSEA Results Summary

  

Fig 1: Enrichment plot: GOBP\_POSITIVE\_REGULATION\_OF\_GLYCOLYTIC\_PROCESS      
 Profile of the Running ES Score & Positions of GeneSet Members on the Rank Ordered List

  

| SYMBOL | TITLE | RANK IN GENE LIST | RANK METRIC SCORE | RUNNING ES | CORE ENRICHMENT || 1 | PFKFB4 | na | 3755 | 0.034 | -0.3097 | No |
| 2 | APP | na | 4058 | -0.002 | -0.3347 | No |
| 3 | HTR2A | na | 4885 | -0.091 | -0.3948 | No |
| 4 | HIF1A | na | 5064 | -0.109 | -0.3990 | No |
| 5 | PSEN1 | na | 5826 | -0.182 | -0.4447 | No |
| 6 | ARNT | na | 6240 | -0.228 | -0.4570 | No |
| 7 | ZBTB20 | na | 7387 | -0.350 | -0.5185 | No |
| 8 | PRKAA2 | na | 7484 | -0.360 | -0.4915 | No |
| 9 | P2RX7 | na | 7528 | -0.364 | -0.4596 | No |
| 10 | ENTPD5 | na | 7783 | -0.394 | -0.4425 | No |
| 11 | MLXIPL | na | 8034 | -0.426 | -0.4219 | No |
| 12 | PRKAA1 | na | 11327 | -1.049 | -0.5945 | Yes |
| 13 | INSR | na | 11461 | -1.101 | -0.4985 | Yes |
| 14 | SLC4A4 | na | 11588 | -1.165 | -0.3958 | Yes |
| 15 | PFKFB2 | na | 11692 | -1.235 | -0.2844 | Yes |
| 16 | IGF1 | na | 11859 | -1.426 | -0.1596 | Yes |
| 17 | PFKFB3 | na | 11970 | -1.771 | 0.0033 | Yes |
Table: GSEA details [plain text format]

  

Fig 2: GOBP\_POSITIVE\_REGULATION\_OF\_GLYCOLYTIC\_PROCESS      
 Blue-Pink O' Gram in the Space of the Analyzed GeneSet

  

Fig 3: GOBP\_POSITIVE\_REGULATION\_OF\_GLYCOLYTIC\_PROCESS: Random ES distribution      
 Gene set null distribution of ES for **GOBP\_POSITIVE\_REGULATION\_OF\_GLYCOLYTIC\_PROCESS**
